# Remimazolam-remifentanil causes less postoperative nausea and vomiting than remimazolam-alfentanil during hysteroscopy: a single-centre randomized controlled trial

**DOI:** 10.1186/s12871-023-02164-3

**Published:** 2023-06-12

**Authors:** Xiaoqiang Zhang, Shuang Li, Jing Liu

**Affiliations:** Department of Anaesthesiology, Mengcheng County No. 1 People’s Hospital, Mengcheng, 233500 Anhui Province P.R. China

**Keywords:** Anaesthesia, Remimazolam, Remifentanil, Alfentanil, Postoperative nausea and vomiting, Hysteroscopy

## Abstract

**Background:**

Although the operation time of hysteroscopy is short, the incidence of postoperative nausea and vomiting is high. The aim of this study was to compare the incidence of postoperative nausea and vomiting in hysteroscopy when remimazolam is combined with remifentanil or alfentanil.

**Methods:**

We conducted a randomized, controlled, double-blind trial. Patients undergoing hysteroscopy were recruited and randomly assigned to either the remimazolam-remifentanil (Group RR) or the remimazolam-alfentanil group (Group RA). All patients in the two groups were started with an induction dose of remimazolam besylate 0.2 mg/kg and then maintained with a dosage of 1.0 mg/kg/h. After induction with remimazolam besylate, in Group RR, remifentanil was infused using a target-controlled infusion system with a target concentration of 1.5 ng/ml and titrated throughout the procedure. In Group RA, infusion of alfentanil was started with an initial bolus dose of 20 µg/kg over 30 s and then maintained at an initial rate of 0.16 µg/kg/min. The primary observation outcome was the incidence rate of postoperative nausea and vomiting. The secondary observation outcomes were the time to awakening, the length of stay in the PACU, the total remimazolam dose and adverse effects, such as low SpO_2_, bradycardia, hypotension and body movement.

**Results:**

A total of 204 patients were successfully included in this study. The incidence of postoperative nausea and vomiting in Group RR (2/102, 2.0%) was significantly lower than that in Group RA (12/102, 11.8%) (p < 0.05). There was no significant difference in the incidence of adverse events, such as low SpO_2_, bradycardia, hypotension and body movement, between Groups RR and RA (p > 0.05).

**Conclusions:**

Remimazolam-remifentanil causes less postoperative nausea and vomiting than remimazolam-alfentanil in hysteroscopy.

**Trial registration:**

Clinical trial registration number: ChiCTR2100044177. Full date of the first registration: 12/03/2021.

**Supplementary Information:**

The online version contains supplementary material available at 10.1186/s12871-023-02164-3.

## Background

The number of operations performed using minimally invasive surgical techniques under general anaesthesia has steadily increased, which has many advantages, but postoperative nausea and vomiting (PONV) remains one of the most common complications [1, 2]. Apfel et al. found that the four risk factors of PONV included in the simple sum score were the female sex, a prior history of motion sickness or PONV, nonsmoking, and the use of postoperative opioids. If none, one, two, three or four of these risk factors were present, the incidence of PONV was 10, 21, 39, 61 and 79%, respectively [3]. Although the operation time of hysteroscopy is short, the incidence of PONV is high [4].

Short-acting opioids such as remifentanil and alfentanil are widely used in fast-track surgeries and hysteroscopy [5–8]. Remimazolam is a new type of benzodiazepine drug that produces sedation and amnesia and is widely used preoperatively, in endoscopic anaesthesia and general anaesthesia induction maintenance, and in intensive care units [9]. Research has shown that compared with propofol, remimazolam besylate combined with remifentanil is a safer anaesthetic substitute in hysteroscopy [10]. However, there is a lack of research on the impact of remimazolam combined with opioids on the incidence of PONV.

The aim of this study was to compare the incidence of PONV in hysteroscopy when remimazolam is combined with remifentanil or alfentanil.

## Methods

### Ethics approval and trial registration

This study was approved by the Clinical Research Ethics Committee of Mengcheng County No. 1 People’s Hospital (2021MYL21003) and was registered with the Chinese Clinical Trial Registry (https://www.chictr.org.cn) on 12/03/2021. The registration number was ChiCTR2100044177. Written informed consent was obtained from the eligible participants in the ward the night before hysteroscopy at Mengcheng County No. 1 People’s Hospital from 15/08/2021 to 20/06/2022.

### Patient inclusion and exclusion criteria

The inclusion criteria were as follows: (1) age between 18 and 65 years; (2) American Society of Anesthesiologists (ASA) physical status I or II; and (3) body mass index (BMI) of 19 to 30 kg/m^2^. The exclusion criteria were as follows: (1) history of alcoholism; (2) allergy to general anaesthetic drugs; (3) presence of renal or liver disease; (4) difficulty with communication; (5) current lactation; and (6) respiratory infection within a week.

### Randomization

Patients were randomly assigned to the remimazolam-remifentanil group (Group RR) or the remimazolam-alfentanil group (Group RA) by a computer-generated random assignment sequence created by an independent researcher using Excel 2010 (Microsoft Office) with two sets of assignments and random block sizes.

### Preoperative preparation and monitoring

All patients fasted routinely 8–12 h before surgery. On arrival in the operating room, a Bene View N15 monitor (Mindray Biomedical Electronics Co., Shenzhen, China) was connected to monitor the electrocardiogram (ECG), noninvasive blood pressure (NIBP) including systolic blood pressure (SBP) and diastolic blood pressure (DBP), heart rate (HR), respiratory rate (RR) and SpO_2_. The SedLine™ monitor (Masimo SedLine®, Masimo Co., US)-derived patient state index (PSI) was used to follow the depth of sedation [11–13]. All patients inhaled oxygen (2 L/min) through nasal oxygen prongs before anaesthesia induction.

### Grouping and intervention

All patients in both Group RR and Group RA were started with an induction dose of remimazolam besylate (Yichang Humanwell Pharmaceutical Co., Ltd., China) 0.2 mg/kg, followed by a maintenance dosage of 1.0 mg/kg/h by continuous IV infusion until the loss of consciousness (absence of eyelash reflex) [10, 14]. When the PSI was ≤ 58 [11–13], hysteroscopy was started. If the PSI was > 58, supplemental remimazolam was added at 2.5 mg/dose, with no more than 5 doses administered within 15 min, according to the instructions of the supplemental drug programme [15].

In Group RR, after induction with remimazolam besylate and confirmation of the absence of the eyelash reflex, infusion with remifentanil (Yichang Humanwell Pharmaceutical Co., Ltd.) was started with a TCI pump (Guangxi VERYARK Technology Co., Ltd., China), and the effective effect-site concentration (Ce) (Minto pharmacokinetic model) was 1.5 ng/ml [16]. Remifentanil was increased by 0.5 ng/ml when analgesia was insufficient (facial grimace, movement, SBP > 140 mmHg, HR > 100 beats/min (bpm) or sudden increase of more than 30 bpm over baseline) and was decreased by 0.5 ng/ml with signs of excessive analgesia (respiratory depression, hypotension, or bradycardia) [17].

In Group RA, after induction with remimazolam besylate and confirmation of the absence of the eyelash reflex, infusion with alfentanil (Yichang Humanwell Pharmaceutical Co., Ltd.) was started with an initial bolus dose of 20 µg/kg over 30 s and then maintained at an initial rate of 0.16 µg/kg/min [8]. The infusion rate of opioids was adjusted according to clinical needs: if the HR or BP increased by more than 30% compared with the level after induction, 10 µg/kg alfentanil was added. This process was repeated if necessary. If the HR and BP remained unchanged for 20 min or decreased, the opioid infusion rate of alfentanil was decreased by 0.8 µg/kg/min [18].

### Outcomes

#### Primary outcome

The primary outcome of this study was the incidence of PONV. All patients in the PACU were continuously observed, and PONV was recorded by a dedicated person in the case of vomiting or active expression of nausea; however, there was no inductive questioning of the patients.

#### Secondary outcomes

The time to awakening, the length of stay in the PACU and the total remimazolam dose were recorded as the secondary outcomes.

The incidence of adverse events, such as low SpO_2_ (intraoperative SpO_2_ ≤ 95%), bradycardia (intraoperative HR < 55 bpm), hypotension (intraoperative SBP < 90 mmHg), and body movement (visible hand bending or head movement) was also recorded. These events were treated by injecting ephedrine or atropine intravenously or through mask ventilation.

Patient data fluctuations included the mean arterial pressure (MAP) (MAP = (SBP + 2 × DBP)/3), HR, RR, SpO_2_, and PSI before anaesthesia (T0), at 2 min post induction (T1), at cervical dilatation (T2), at the end of the operation (T3), and at awakening (T4).

### Sample size and statistical analysis

SPSS statistics 17.0.1 (SPSS, Inc., Chicago, Illinois) was used for statistical analysis. For data analysis, the normality test in SPSS statistical software was used to determine whether the data conformed to a normal distribution. Continuous variables with a normal distribution are expressed as the mean ± standard deviation and were analysed by Student’s t test. The Mann‒Whitney U test was used for continuous variables with a nonnormal distribution. Haemodynamic parameters were compared with repeated ANOVA measurements. Categorical variables are expressed as frequencies (percentages) and were analysed using the Pearson chi-square test. The Wilcoxon signed-rank test was used to compare continuous variables. A p value < 0.05 was considered statistically significant.

Because the incidence of PONV caused by different risk factors varies, we assumed that the incidence rate of PONV would be decreased from 39 to 19% in this study. A sample size of 102 participants in each group was calculated, and the significance level was 0.05 (α = 0.05). Given a 10% attrition rate, the strength was 80% (β = 0.20) [19].

## Results

**Fig. 1:**
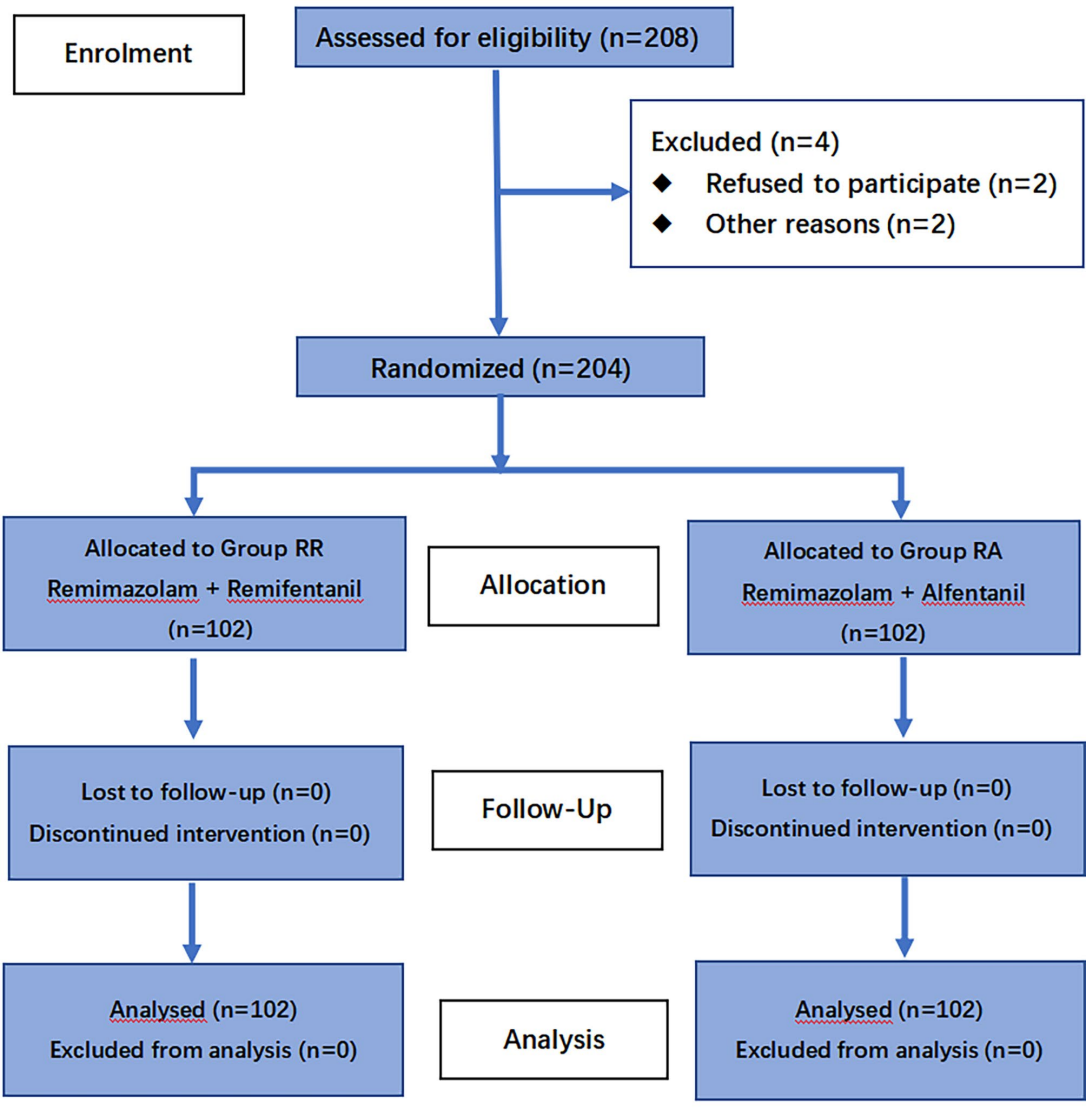
CONSORT diagram of patient recruitment

The study population comprised 204 randomly coded patients in Group RR (n = 102) and Group RA (n = 102) (Fig. 1).

The demographic and surgical characteristics of the patients are listed in Table 1. The characteristics of patients in the two groups were similar.

**Table 1:** Demographic characteristics for each group

	Group RR (n = 102)	Group RA (n = 102)	p value
Age, mean ± SD, year	43.3 ± 7.7	42.28 ± 8.4	0.692
Height, mean ± SD, cm	159.2 ± 4.7	158.4 ± 4.6	0.691
Weight, mean ± SD, kg	62.8 ± 7.8	61.6 ± 7.0	0.256
BMI, mean ± SD, kg/m^2^)	24.7 ± 2.8	24.5 ± 2.4	0.073
ASA, n(%)			
I	97(84.3)	86(95.1)	
II	5(15.7)	16(4.9)	
Duration of operation, mean ± SD, min	9.6 ± 4.2	8.9 ± 3.7	0.241
Total alfentanil, mean ± SD, ug	---	894.4 ± 194.2	

Note: *ASA* American Society of Anesthesiologists, *BMI* body mass index, *SD* standard deviation, *Group RR* remimazolam-remifentanil group, *Group RA* remimazolam-alfentanil group

PONV occurred on 2 (2.0%) occasions in group RR and 12 (11.8%) occasions in group RA (p = 0.006), and spontaneously resolved without drug treatment in all cases in the two groups (Table 2). In this study, both groups of patients had an Apfel score of more than 2 in the PONV risk assessment, representing a medium to high risk of PONV.

**Table 2:** Primary and secondary outcomes for each group

	Group RR (N = 102)	Group RA (n = 102)	p value
Primary outcome			
PONV n(%)	2 (2.0%)	12 (11.7%)	0.006*
Secondary outcomes			
Time to awakening, mean ± SD, s	200.6 ± 94.6	283.5 ± 183.1	< 0.001*
PACU length of stay, mean ± SD, s	354.1 ± 66.8	371.8 ± 67.0	0.639
Total remimazolam, mean ± SD, mg	22.3 ± 4.7	21.7 ± 4.5	0.722
Adverse events, n(%)			
Low SpO_2_	15 (14.7%)	22 (21.6%)	0.203
Bradycardia	3 (2.9%)	3 (2.9%)	1.000
Hypotension	8 (7.8%)	10 (9.8%)	0.622
Body movement	30 (29.4%)	27 (26.5%)	0.640
Total remifentanil, mean ± SD, µg	69.1 ± 21.6	---	

Note: *PONV* Postoperative nausea and vomiting, *SD* Standard deviation, *PACU* post anaesthesia care unit, *Group RR* remimazolam-remifentanil group, *Group RA* remimazolam-alfentanil group

The time to awakening in Group RR (200.6 ± 94.6 s) was significantly shorter than that in Group RA (283.5 ± 183.1 s) (p < 0.001). However, there was no significant difference in the length of stay in the PACU or the total remimazolam dose between Groups RR and RA. There was no significant difference in the incidence of adverse events, such as low SpO_2_, bradycardia, hypotension and body movement, between Groups RR and RA.

**Fig. 2:**
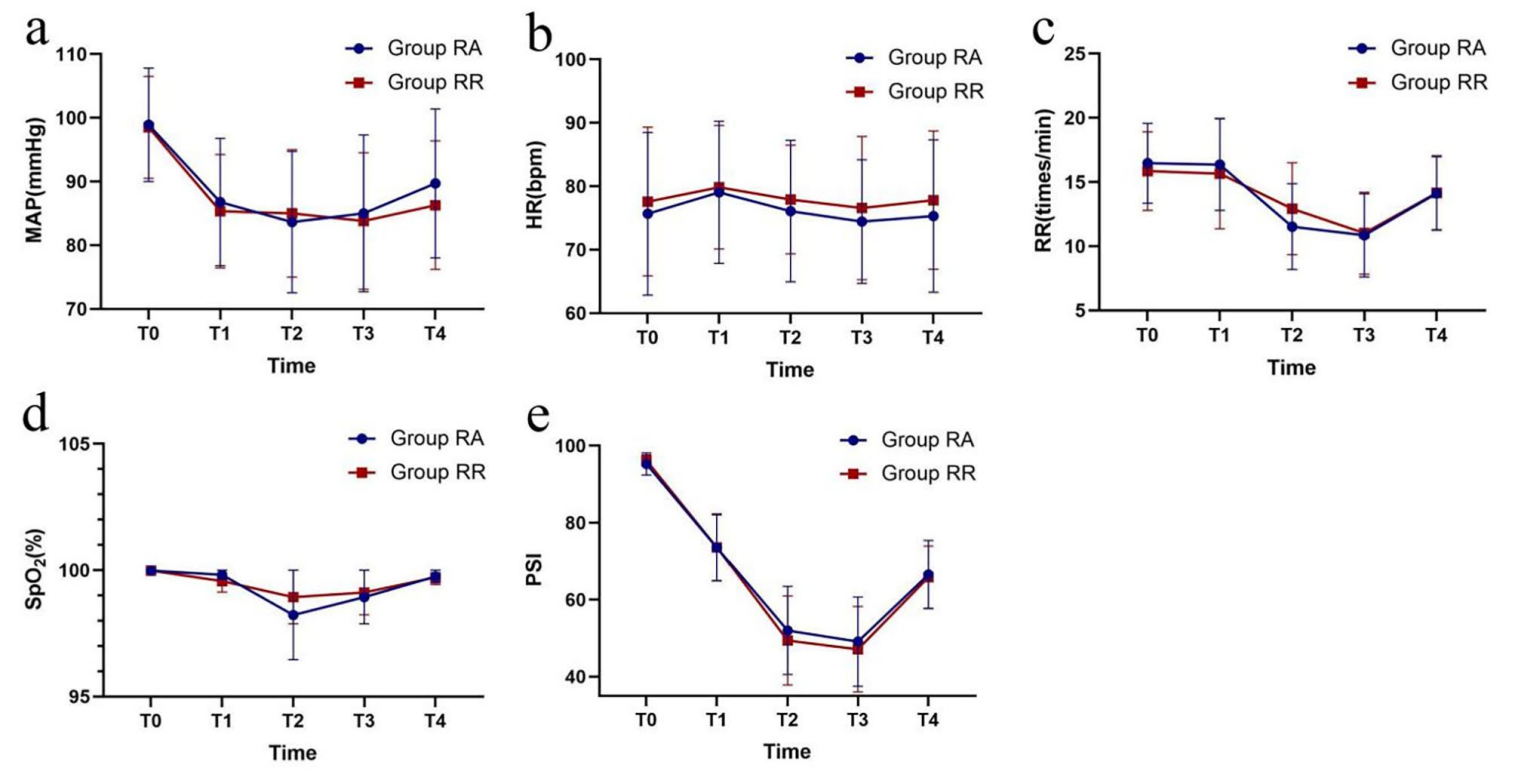
Changes in MAP, HR, RR, SpO_2_ and PSI Changes in MAP (**a**), HR (**b**), RR (**c**), SpO_2_ (**d**) and PSI (**e**). MAP, HR: Normal distribution, mean, and SD. SpO_2_, RR, PSI: Nonnormal distribution, median, and upper/lower limit. T0, before anaesthesia, T1, at 2 minutes post induction, T2, at cervical dilatation, T3, at the end of the operation, and T4, at awakening. Group RR, remimazolam-remifentanil group, Group RA, remimazolam-alfentanil group. The two groups showed similar MAP, HR, RR and SpO_2_ values with little fluctuation. During hysteroscopy, the PSI values in both groups were similar and showed sufficient and effective anaesthesia depth

Compared with those at T0, the MAP, HR, RR and SpO_2_ were all reduced in the two groups at T1-4 (Fig. 2). The two groups showed similar MAP, HR, RR and SpO_2_ values, with little fluctuation.

### PSI

To achieve a more objective determination of the depth of conscious sedation than the MOAA/S score, we adopted the method of monitoring the PSI. Previous studies have confirmed that there is a significantly strong correlation between the PSI and both the Ramsay and OAAS scales [20, 21]. In our study, all selected patients successfully underwent sedation anaesthesia and hysteroscopy. No patients in either group needed further medication or withdrew from this study due to insufficient anaesthesia depth.

During hysteroscopy, the PSI values in both groups were similar and showed sufficient and effective anaesthesia depth (Fig. 2).

## Discussion

Our trial compared the incidence of PONV between remimazolam-remifentanil and remimazolam-alfentanil in hysteroscopy. Based on our data, remimazolam-remifentanil causes less PONV than remimazolam-alfentanil in hysteroscopy.

During the period of this study, we did not observe any serious side effects in either patient group or side effects requiring withdrawal from this study. The incidence of PONV in Group RR (2/102, 2.0%) was significantly lower than that in Group RA (12/102, 11.8%) (p < 0.05). There was no significant difference in the incidence of adverse events, such as low SpO_2_, bradycardia, hypotension and body movement, between Groups RR and RA. The two groups showed similar MAP, HR, RR and SpO_2_ values, with little fluctuation.

In previous research by Yi et al., the incidence of PONV in the PACU after anaesthesia with remimazolam combined with alfentanil reached 50% [7]. Their result differs greatly from the results observed in our study. Their research protocol has some differences from ours such as the dosage of alfentanil was relatively high, used muscle relaxants, tracheal intubation was performed, long surgical time, etc. However, whether these differences contributed to differences in experimental results requires further research.

Remimazolam is a new ultra–short-acting benzodiazepine and has the advantages of a rapid onset, high clearance rate (1.14 L/min), and metabolic process that is independent of liver and kidney function [21]. In a study by Hari et al., compared to general anaesthesia with desflurane, anaesthesia with remimazolam can reduce the incidence of PONV following laparoscopic gynaecological surgery [22]. Research has confirmed that the prophylactic administration of midazolam reduced the incidence of PONV [23]. Similarly, based on the low incidence of PONV in our study, whether remimazolam can also reduce the incidence of PONV, similar to midazolam, requires further research. When dexmedetomidine or ketamine is used with opioids, the incidence of PONV can be reduced to some extent [24, 25]. More clinical trials are needed to investigate the differences in efficacy, incidence of PONV, and incidence of other side effects with the use of remimazolam and other categories of sedative drugs.

Previous studies have compared the incidence of PONV between remifentanil and alfentanil [26, 27]. Due to differences in research methods, types of surgery, drug combination and other aspects, the results of these comparisons of PONV between remifentanil and alfentanil are also different. Remifentanil and alfentanil are both ultra - short - acting opioid drugs, but there are subtle differences in pharmacokinetics and pharmacodynamics. The central clearance rate of remifentanil is significantly higher than alfentanil(2.9 vs. 0.36 L/min), and the terminal half-life of remifentanil is 35.1 min, while that of alfentanil is 94.5 min [28, 29]. Remimazolam is a new ultra–short-acting benzodiazepine wich have a high clearance rate (1.14 L/min) [21]. Regarding the difference in the incidence of PONV in our study, we speculate that the reason is that remimazolam and remifentanil were cleared synchronously; however, remimazolam and alfentanil cannot be cleared synchronously after drug withdrawal. Further research is needed to determine whether the differences in the pharmacokinetics and pharmacodynamics of the two drugs lead to differences in the incidence of PONV and whether they can have similar effects in longer surgeries or other operations.

There are some limitations to this study. This was a single-centre investigation, which limited the statistical analysis of the two groups of patients. Because remimazolam is a newly marketed drug, to ensure the safety of patients, we selected populations with low risk when designing the inclusion criteria for the trial, and the conclusion should be interpreted with caution. Additionally, we only evaluated the occurrence of PONV in the PACU and did not evaluate the occurrence of PONV within 24 h after surgery. Our main concern in this study was the incidence of PONV, but we did not record arbitrary values on a numerical rating scale (NRS) ranging from 0 (“everything okay”) to 10 (“vomiting”) to assess the degree of PONV [30]. More comprehensive studies are needed to evaluate the incidence of PONV in this context.

## Conclusions

Remimazolam-remifentanil causes less PONV than remimazolam-alfentanil in hysteroscopy. This was a single-centre study that had some limitations, and multicentre studies are recommended to obtain more relevant conclusions.

## Electronic supplementary material

Below is the link to the electronic supplementary material.

Supplementary Material 1

Supplementary Material 2

## Data Availability

The datasets generated and analysed during the current study are not publicly available due to institutional restrictions but are available from the corresponding author on reasonable request.
