# Supplementary material for: Remimazolam-remifentanil causes less postoperative nausea and vomiting than remimazolam-alfentanil during hysteroscopy: a single-centre randomized controlled trial
Source: BMC Anesthesiol. 2023 Jun 12;23:199. doi: 10.1186/s12871-023-02164-3 (PMC10259043; doi:10.1186/s12871-023-02164-3)
Supplement: Supplementary file 1 — Supplementary Material 1 [file 12871_2023_2164_MOESM1_ESM.docx]

**Table .** Adverse events at different time points for each group.

|  | intraoperative | | during anesthesia | | post-induction | |
| --- | --- | --- | --- | --- | --- | --- |
|  | Group RR | Group RA | Group RR | Group RA | Group RR | Group RA |
| Low SpO_2_ | 2(2.0%) | 5(4.9%) | 6(5.9%) | 8(7.8%) | 6(5.9%) | 9(8.8%) |
| Bradycardia | 0 | 0 | 1(1.0%) | 1(1.0%) | 2(2.0%) | 2(2.0%) |
| Hypotension | 1(1.0%) | 1(1.0%) | 2(2.0%) | 3(2.9%) | 5(4.9%) | 6(5.9%) |
| Body movement | 28(27.5%) | 26(25.5%) | 2(2.0%) | 1(1.0%) | 0 | 0 |

Note:Values are presented as n (%), *Group RR* remimazolam-remifentanil group, *Group RA* remimazolam-alfentanil group.
